# Anticancer Effects of Secoiridoids—A Scoping Review of the Molecular Mechanisms behind the Chemopreventive Effects of the Olive Tree Components Oleocanthal, Oleacein, and Oleuropein

**DOI:** 10.3390/nu16162755

**Published:** 2024-08-18

**Authors:** Ikhwan Yuda Kusuma, Habibie Habibie, Muh. Akbar Bahar, Ferenc Budán, Dezső Csupor

**Affiliations:** 1 Institute of Clinical Pharmacy, University of Szeged, 6725 Szeged, Hungary; ikhwanyudakusuma@uhb.ac.id (I.Y.K.); akbarbahar@unhas.ac.id (M.A.B.); 2 Pharmacy Study Program, Universitas Harapan Bangsa, Purwokerto 53182, Indonesia; 3 Department of Pharmacy, Faculty of Pharmacy, Hasanuddin University, Makassar 90245, Indonesia; habibie@unhas.ac.id; 4 Institute of Physiology, University of Pécs, 7624 Pécs, Hungary; 5 Institute for Translational Medicine, University of Pécs, 7624 Pécs, Hungary

**Keywords:** olive oil, olive tree, *Olea europaea*, oleocanthal, oleuropein, oleacein, chemoprevention, cancer

## Abstract

The olive tree (*Olea europaea*) and olive oil hold significant cultural and historical importance in Europe. The health benefits associated with olive oil consumption have been well documented. This paper explores the mechanisms of the anti-cancer effects of olive oil and olive leaf, focusing on their key bioactive compounds, namely oleocanthal, oleacein, and oleuropein. The chemopreventive potential of oleocanthal, oleacein, and oleuropein is comprehensively examined through this systematic review. We conducted a systematic literature search to identify eligible articles from Scopus, PubMed, and Web of Science databases published up to 10 October 2023. Among 4037 identified articles, there were 88 eligible articles describing mechanisms of chemopreventive effects of oleocanthal, oleacein, and oleuropein. These compounds have the ability to inhibit cell proliferation, induce cell death (apoptosis, autophagy, and necrosis), inhibit angiogenesis, suppress tumor metastasis, and modulate cancer-associated signalling pathways. Additionally, oleocanthal and oleuropein were also reported to disrupt redox hemostasis. This review provides insights into the chemopreventive mechanisms of *O. europaea*-derived secoiridoids, shedding light on their role in chemoprevention. The bioactivities summarized in the paper support the epidemiological evidence demonstrating a negative correlation between olive oil consumption and cancer risk. Furthermore, the mapped and summarized secondary signalling pathways may provide information to elucidate new synergies with other chemopreventive agents to complement chemotherapies and develop novel nutrition-based anti-cancer approaches.

## 1. Introduction

### 1.1. Global Burden of Cancer

With 9.7 million cases of cancer-related fatalities worldwide in 2022, it is the second most common cause of death overall after cardiovascular diseases [1]. Between 2020 and 2050, the worldwide economic burden of cancer is estimated at USD 25.2 trillion (using constant 2017 prices). This constitutes 0.55% of the global gross domestic product [2]. In the year 2019, it was estimated that 250 million disability-adjusted life years (DALYs) were due to cancer, which manifests a 16.0% increase since 2010 [3].

Novel chemopreventive techniques are therefore necessary to enhance anti-carcinogenic therapies as well as review the known mechanism to combine beneficial compounds to utilize their synergies as food supplements or functional foods. Here, we update the review of R. Fabiani focusing on both in vivo and in vitro effects of the secoiridoid content of olive oil such as oleocanthal, oleacein, and oleuropein [4]. Also, we complement here in our systematic review the narrative review of Emma RM. et al. about the preclinical effects of the most relevant secoiridoids [5].

### 1.2. The History and Compounds of Olive Oil

The olive tree (*Olea europaea*, Oleaceae) and the olive oil produced from its fruit are an integral part of European culture. Olive trees have been growing for many seasons and the oil extracted from the fruit has been used since the Neolithic age for various purposes, mainly as food [6]. The beneficial fatty acid and water-soluble components of olive oil have long been known [7]. The Seven Countries Study, begun in the 1950s, showed a negative correlation between all-cause mortality, and olive oil consumption, which was attributed to the high levels of monounsaturated fatty acids in olive oil. Speculatively, the effects were largely attributed to the main fatty acid component, oleic acid [8]. However, nowadays, a high number of health-promoting components are known in olive oil. The extra virgin olive oil is the most abundant in such molecules as triglycerides (97–99%) as well as minor compounds (1–3%). The fatty acid components of olive oil triglycerides comprise monounsaturated fatty acids (MUFA, 65–83%), especially oleic acid, and in smaller quantity polyunsaturated fatty acids (PUFA), for example linoleic acid. There are also phenolic compounds such as phenolic alcohols, tyrosol (p-hydroxyphenylethanol; p-HPEA) and hydroxytyrosol (3,4-dihydroxyphenylethanol; 3,4-DHPEA), together with their secoiridoid derivatives p-HPEA-EA (ligstroside aglycone), 3,4-DHPEA-EA (oleuropein aglycone), 3,4-DHPEA-EDA (oleacein), and p-HPEA-EDA (oleocanthal). Furthermore, tocopherols and other compounds such as hydrocarbons (for example squalene) and provitamin A compounds are worthy of mentioning [5,9]. 

### 1.3. Epidemiological Data about the Anti-Cancer Effects of Olive Oil Consumption

A study showed that olive oil consumption is associated with a reduced risk of lung cancer (OR: 0.65; *p* < 0.05; 95% CI: 0.42–0.99) [10]. Pelucchi et al. conducted a meta-analysis that found a significantly lower risk of breast cancer in a group who consumed a diet rich in olive oil (highest quartiles of the examined population with daily > 28.05 g intake) compared to persons with the lowest olive oil consumption (lowest quartiles with daily < 9.35 g intake) (pooled RR 0.62; 95% CI 0.44–0.88) [11]. Additionally, a case–control study demonstrated a protective effect against laryngeal cancer in persons who consumed higher amounts of olive oil (highest quartile) compared to those who consumed less (lowest quartile) (OR: 0.4; *p* = 0.01; 95% CI: 0.3–0.7) [12]. According to the meta-analysis of Markellos et al., the high quantity of olive oil consumption compared to the lowest quantity was associated with a 31% lower likelihood (pooled RR = 0.69) of cancer (95% CI: 0.62–0.77) [13]. Lastly, a dose-dependent reverse relationship was found between olive oil consumption and the risk of bladder cancer (lowest tertile compared to the highest tertile associated with a lower risk) (OR: 0.47, *p*-trend = 0.002; 95% CI: 0.28–0.78) [14].

### 1.4. The Relevance of Secoiridoids

Subsequent research has shown that polyunsaturated fatty acids, especially omega-3 fatty acids, play a greater role in health maintenance than monounsaturated fatty acids. These fatty acids are metabolized in the body into various prostanoids (e.g., alpha-linolenic acid to prostacyclin), which play an important role in cardiovascular prevention through their anti-inflammatory and antithrombotic effects. However, oils rich in omega-3 fatty acids appear to be less relevant for prevention. Although rapeseed oil, walnut oil, and soybean oil are significantly higher in omega-3 fatty acids than olive oil [15], their cardiovascular preventive effects are less well suggested. Other oils with a high oleic acid content similar to that of olive oil (peanuts, avocados) [15] have not been found to have similar effects to olive oil. Of course, this may be partly explained by the fact that, unlike olive oil, these oils are not consumed regularly by large populations, which makes epidemiological observations difficult, but the preclinical results with olive oil are also more favorable than with other oils. In animal experiments, feeding with olive oil resulted in decreased platelet hyperactivity and subendothelial thrombogenicity [15]. In a clinical trial, it was observed that, unlike olive oil, rapeseed and sunflower oils did not have an antithrombotic effect [16]. This suggests that other components, in addition to the fatty acids, are involved in the beneficial effects of olive oil. This hypothesis is further reinforced by the fact that no similar advantageous effects have been described for non-extra virgin olive oils with the same fatty acid composition as this extra virgin olive oil. The mentioned minor components in extra virgin olive oils are absent in other olive oils as a result of refining processes [17].

**Figure 1:**
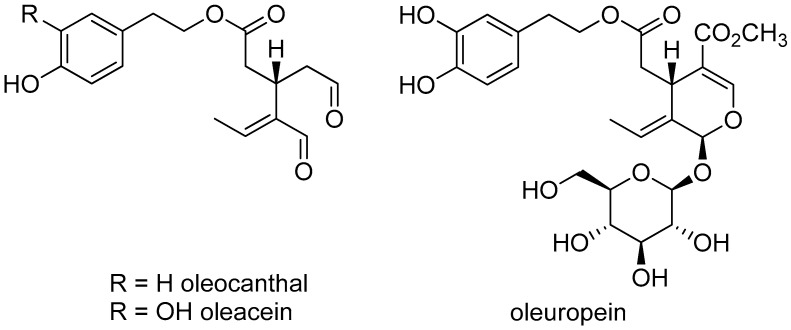
The major secoiridoids of *O. europaea*.

A breakthrough in understanding the mechanism of action of olive oil came with the discovery of oleocanthal (Figure 1). This secoiridoid was described in 1993 [18], but its biological importance was only later recognized. The anti-inflammatory activity of oleocanthal was documented in 2005, and this bioactivity plays an important role in antithrombotic and cardiovascular preventive effects. Interestingly, oleocanthal was identified in a study looking for a substance with a flavor similar to ibuprofen in olive oil [19].

The second most quantitatively important secoiridoid in olive oil, oleacein, was identified in a bioassay-guided study to identify compounds that inhibit the angiotensin convertase enzyme [20]. Although oleocanthal is found in concentrations of approximately 300 mg/kg, oleacein is found in approximately a third of this amount in extra virgin olive oil. In non-extra virgin olive oil, the two compounds are found at an order of magnitude lower concentration [21]. 

Oleacein was first discovered not from olive oil but from olive leaf extract [20]. The main secoiridoid of olive leaves, oleuropein, was discovered several decades earlier [22]. Secoiridoids may not only be related to the beneficial effects of olive oil shown in epidemiological studies but may also explain the effects of olive leaf in traditional medicine. The leaves of the olive tree have been widely used in folk medicine in the Mediterranean region to reduce fever of different origin, including malaria. Among its other medicinal uses, the leaf is thought to have an antihypertensive and diuretic effect [21]. According to the monograph of the European Medicines Agency, olive leaf can be marketed as traditional herbal medicinal products to promote renal elimination of water in mild cases of water retention [23]. 

Oleocanthal, oleacein, and oleuropein are secoiridoids characteristic of *O. europea*. In addition to their anti-oxidant and anti-inflammatory effects, these compounds may also have bioactivities that may play a role in the beneficial effects observed in cell cultures, animal experiments, epidemiological or clinical studies of olive oil or olive leaf [24,25]. 

The aim of our work is to systematically review and summarize the literature on the mechanisms of chemopreventive effects of the main secoiridoids of *O. euopaea*, namely oleocanthal, oleacein and oleuropein.

## 2. Materials and Methods

### 2.1. Study Design

We conducted a systematic literature search to identify eligible scientific articles published between established articles up to 10 October 2023. This scoping review was reported according to the Preferred Reporting Items for Systematic reviews and Meta-Analyses extension for Scoping Review (PRISMA-ScR) guidelines [14]. All selected studies were imported into the automated citation checking service Zotero (https://www.zotero.org/, accessed on 10 October 2023) to identify and eliminate duplicate records.

### 2.2. Search Strategy

The systematic literature search was performed using Scopus, PubMed, and Web of Science databases on October 10th, 2023. The search key comprised the keywords “oleocanthal” OR “OLE6 protein” OR “Olea europaea” OR “OLE9 protein” OR “Olea europaea” OR “OLE3 protein” OR “Olea europaea” OR “olive leaf extract” OR “Olive Oil” OR “Oleocanthal” OR “Olea europaea” OR “Olive leaf extract” OR “Olive polyphenol” OR “Olive” OR “Olive oil” OR “Extra virgin olive” OR “Olive compound” OR “Olive extract” OR “Olive oil phenolic”) OR (“oleuropein” OR “10-hydroxyoleuropein” OR “Ligustrum” OR “Oleuropein” OR “Olea europaea” OR “Olive leaf extract” OR “Olive polyphenol” OR “Olive” OR “Olive oil” OR “Extra virgin olive” OR “Olive compound” OR “Olive extract” OR “Olive oil phenolic” OR “10-hydroxyoleuropein” OR “Ligustrum”) AND (“Chemoprevention” OR “Chemoprevent*” OR “Anticancer” OR “Antitumor” OR “Tumor prevent*” OR “Cancer chemoprevent*”. The detailed search strategy for each database is provided in Supplementary Material (Supplementary Information S1).

### 2.3. Eligibility Criteria

This review employed specific inclusion and exclusion criteria tailored to the scope of this study. Inclusion criteria encompassed in silico, in vitro, in vivo, or clinical studies involving the main secoiridoids of *O. europaea*, including oleocanthal, oleacein, and oleuropein focusing on their effects and mechanisms of chemoprevention, without language restriction. Exclusion criteria comprised studies reporting only cytotoxic effects against certain cell lines, investigations involving complex mixtures, such as those from olive mill wastewater or oil production waste products, studies focusing on major metabolite of oleuropein such as hydroxytyrosol, and articles such as reviews, book reviews, book chapters, conference abstracts, notes, and communication articles.

### 2.4. Study Selection

Two independent reviewers (IYK, MAB) screened the titles and abstracts of all articles for eligibility employing the predefined inclusion and exclusion criteria using Rayyan AI software [15]. The data extraction screening included information on intervention details, study design, tissue/cell types, mechanism of action, and outcomes. Any disagreements were resolved through discussion and consensus. The full texts of potentially eligible papers were then reviewed by the same two reviewers (IYK, MAB) using the same criteria. A third reviewer (DC) was involved to solve any disagreements during the screening process. Two reviewers (IYK, HH) separately extracted the data then cross-checked by MAB and DC. Using a standardized template in Microsoft Office Excel 2019, the synthesis comprised a thematic analysis of the reported findings and a descriptive overview of the study’s characteristics (see Table 1).

## 3. Results

**Figure 2:**
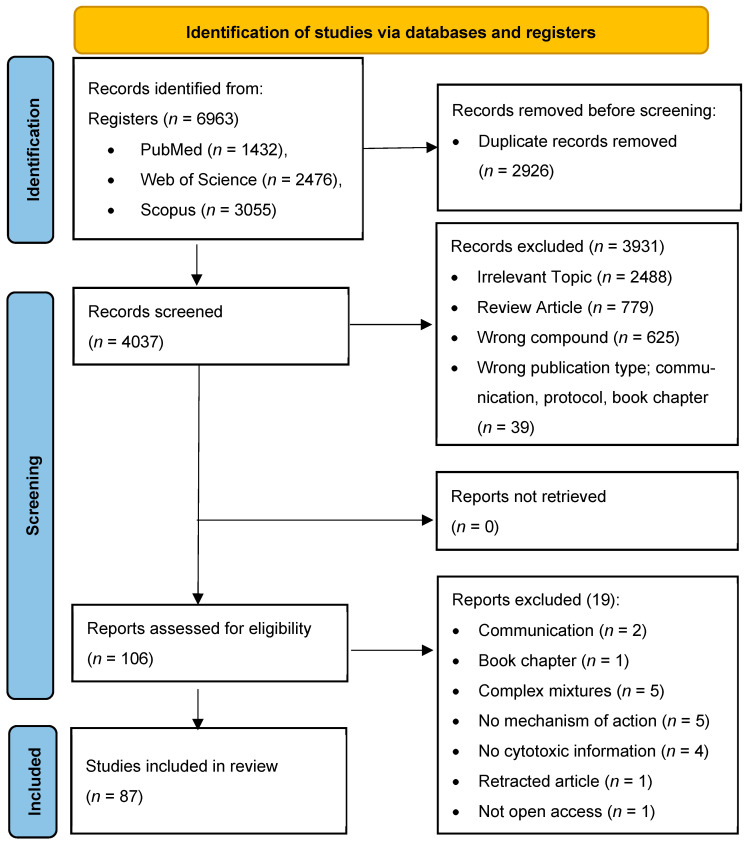
PRISMA flow diagram for identification of relevant studies.

The database search revealed 6963 records for initial review, comprising 1432 from PubMed, 3055 from Scopus, and 2476 from Web of Science. Following the elimination of duplicates (*n* = 2924), there were 4039 articles screened based on titles and abstracts. After the exclusion of irrelevant articles (i.e., not related to the chemopreventive effects of *O. europaea* secoiridoids, *n* = 3929), a total of 110 articles were selected for a comprehensive full-text review. Ultimately, 88 articles met the previously established inclusion criteria (Figure 2).

### 3.1. Chemopreventive Mechanism of Oleocanthal, Oleuropein, and Oleacein

**Figure 3:**
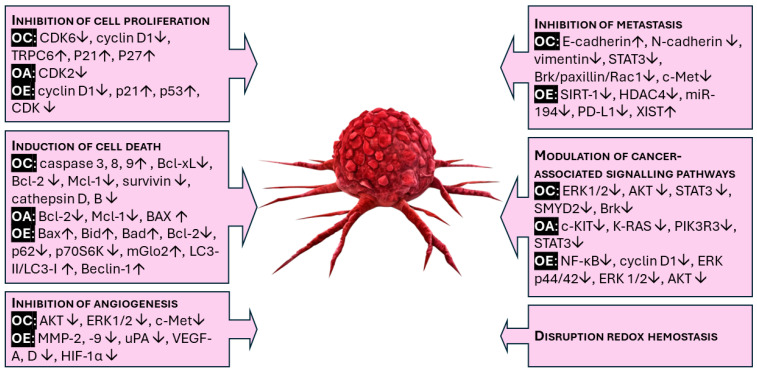
The key elements of the chemopreventive effects of oleocanthal (OC), oleacein (OA), and oleuropein (OE).

Chemoprevention by definition means the prevention of any diseases using medicines or other beneficial materials (which are mostly intaken by nutrition). Nowadays, chemoprevention refers mostly to cancer prevention, namely to hinder or delay the development, progression, or relapse of cancer. The first approved chemopreventive agent was tamoxifen, capable of diminishing the risk of developing estrogen receptor-positive breast cancer. This was succeeded by the second-generation raloxifene, which proved advantageous in preventing breast cancer within high-risk populations [113]. Since then, chemoprevention has emerged as an intriguing focus and avenue for both cancer treatment and prevention. The primary mechanisms of cancer chemopreventive agents involve promoting antioxidant and anti-inflammatory activities, inhibiting cell proliferation via modulating cell cycle arrest, apoptosis, necrosis, and autophagy, regulating signal transduction pathways, as well as inhibiting tumor angiogenesis and metastasis [114]. The mechanisms involved in the chemopreventive effects of oleocanthal, oleacein, and oleuropein are summarized in Figure 3.

#### 3.1.1. Inhibition of Cell Proliferation (Cell Cycle Arrest)

One of the key features of a cancer cell is its ability for continuous proliferation even in the absence of growth stimuli. Cancer cells exhibit deregulated signalling cascades, allowing them operation with varying degrees of independence from proliferation signals, ultimately leading to uncontrolled growth [115]. The cell-cycle machinery exhibits abnormal activity in virtually all types of tumors, serving as a significant driving force behind tumorigenesis leading to uncontrolled proliferation [116]. Numerous chemopreventive agents are recognized for their ability to regulate cell cycle progression [117]. The primary protein regulators governing the cell cycle include cyclins, cyclin-dependent kinases (CDKs), and CDK inhibitors (CKIs). During mitogenesis, cells traverse a restriction point in the G1 phase before advancing into the S phase and subsequently undergoing mitosis. In the early stages of G1 phase progression, mitogenic factors enhance the expression of cyclin D1. This is followed by the binding of cyclin D1 to CDK4/6, forming cyclin/CDK complexes. Subsequently, these complexes deactivate the cell cycle restriction protein, retinoblastoma (Rb), leading to the release of E2F transcription factors, initiating the transcription of progression related genes through the S phase [118]. Upstream inhibitors, such as p21 and p27, modify the activity of the CDK-cyclin complexes.

- Oleocanthal

Oleocanthal has demonstrated a viability inhibitory effect on a wide range of cancer cells, including those associated with breast cancer [26,29,31,39,40,45], liver cancer [32,33], colon cancer [32], pancreatic cancer [26], melanoma [28], prostate cancer [27], lung cancer [38], and neuroblastoma [43]. Oleocanthal has been linked to the interruption of cancer cell progression at different stages of the cell cycle by inducing and inhibiting various protein regulators and checkpoints. For instance, in MDA-MB-231 human breast cancer cells, oleocanthal triggers G1/M arrest by downregulating the expression of CDK6 and cyclin D1 while upregulating p21 and p27 expression, and this is confirmed with mitosis suppression via reduction in proliferation marker (Ki-67) positive staining in the tumor tissue of the MDA-MB-231 xenograft model [39]. Oleocanthal also triggers G0/G1 phase arrest in human hepatocarcinoma cells (HCC) by suppressing cyclin D1 [33], likely via the inhibition of its transcription factor STAT3 [119]. This inhibition results in reduced STAT3 phosphorylation, impeding its nuclear translocation and DNA binding activity [33].

The role of calcium signalling in cell proliferation has been extensively documented. The recurrent calcium spikes essential for this process require the release from internal stores and influx of external calcium. Calcium signal induces resting cells (G0) to re-enter the cell cycle. Furthermore, during the G1/S transition, it may facilitate the initiation of DNA synthesis [120]. In breast cancer cells (MCF7 and MDA-MB-231), oleocanthal inhibits cell growth by decreasing transient influx of Ca^2+^ in a concentration-dependent manner. This is achieved through the silencing of gene expression of the Transient Receptor Potential Canonical 6 (TRPC6) channel, which is a Ca^2+^ transporter [31].

b. Oleacein

In vitro, oleacein has shown a viability inhibitory effect on melanoma [110], neuroblastoma [111], and multiple myeloma [112]. Oleacein induces cell cycle arrest in melanoma cells at the G1/S phase transition, followed by a significant increase in the phosphorylation of Cdk2, a crucial regulator of this transition, at Tyr15. This phosphorylation event renders Cdk2 inactive, as it is typically activated when in its dephosphorylated form [110]. In multiple myeloma cells, oleacein induces cell cycle arrest by upregulating the expression of cell cycle inhibitors p27^KIP1^ and p21^CIP1^ proteins, leading to an increase in the percentage of hypodiploid cells [sub-G0 phase]. Additionally, it induces the accumulation of cells in the G0/G1 phase [112].

c. Oleuropein

Oleuropein induces cell cycle arrest on a wide range of cancer cells, including those associated with breast cancer [48,51,60,81], liver cancer [108], cervical cancer [94], pancreatic cancer [67], lung cancer [80,85], and neuroblastoma [76]. In breast cancer cells, oleuropein triggers the accumulation of cells in the S phase of the cell cycle, suggesting a delay occurring during DNA replication [48,60]. This cell cycle arrest is facilitated by the downregulation of cyclin D1 and the upregulation of p21, which is a cyclin D-dependent kinase inhibitor [48]. InHepG2 cells, oleuropein prompts sub-G1 and G2/M phase arrest, linked to the upregulation of p53-a potent tumor suppressor gene, which is important in apoptosis signalling; it also activates p21, which acts as an inhibitor of G2/M phase-related proteins. Meanwhile, there is a notable reduction in CDK1 and cyclin B1 levels [108].

#### 3.1.2. Induction of Cell Death (Apoptosis, Autophagy, and Necrosis)

The initiation and progression of all cancers are linked to the deregulation of cell proliferation and the inhibition of cell death, particularly apoptosis. In cancer, the insufficient occurrence of apoptosis results in positive selection via increased proliferation of malignant cells. Many chemopreventive substances are acknowledged for their capacity to induce cell death, effectively suppressing tumor growth. Apoptosis is typically carried out via two primary pathways: the death receptor-mediated extrinsic pathway and the mitochondria-mediated intrinsic pathway that leads to caspase activation [121]. In addition to necrosis and apoptosis, autophagy represents another form of programmed cell death relevant in cancer, among others. Autophagy, derived from Greek words meaning “self-eating”, plays a role in the extensive breakdown of long-lived cytosolic proteins and organelles. It serves as an important intracellular process for maintaining health by degrading the damaged or malignantly transformed cells, including cells with premalignant signal transduction [122].

- Oleocanthal

Oleocanthal triggers apoptosis in a broad spectrum of cancer cells [29,32,33,36,43,44,46]. Oleocanthal induces apoptosis via activation of caspase-8, -9 and -3 followed by the cleavage of poly(ADP-ribose) polymerase (PARP) in hepatocellular carcinoma [33], hematopoietic tumor cells [29], and breast cancer [39]. Furthermore, using Z-VAD-FMK, a pan-caspase inhibitor, oleocanthal was confirmed to induce apoptosis partially via caspase-dependent apoptosis in melanoma cells (A375) [44] and completely via caspase-dependent apoptosis in breast cancer cells (MDA-MB-231) [39]. Oleocanthal activates a mitochondrial-mediated intrinsic pathway via the upregulation of reactive oxygen species (ROS) and mitochondrial membrane depolarization [29,32]. Moreover, oleocanthal downregulates the expression of antiapoptotic protein such as Bcl-xL, Mcl-1, Bcl-2, survivin in melanoma and hepatocarcinoma cells [28,33,44]. This results in heightened mitochondrial permeability, leading to the release of cytochrome c into the cytoplasm [123] and subsequently triggering the activation of caspase-3 through the formation of the apoptosome [124]. Interestingly, the apoptosis-inducing effect of oleocanthal extends beyond its ability to modulate antiapoptotic proteins. Oleocanthal in 20 µmol concentration also triggers lysosome-dependent cell death across various cancer cell lines, namely PC3 human prostate cancer cells, MDA-MB-231 human breast cancer cells, N134 murine PNET cancer cells, and BxPC3 pancreatic cancer cells. In contrast, the viability of normal cells, such as HEK293T human kidney cells, MCF10A human breast cancer cells, and BJ-hTERT human fibroblast cells, were not afflicted [26,36]. It induces lysosomal membrane permeabilization marked by robust galectin-3 translocation to lysosomes [26], leading to the release of degradative enzymes from lysosomes, such as cathepsins (cathepsin D and B), into the cytosol [26,125]. Depending on the degree of lysosomal membrane permeabilization, it can induce both apoptotic and non-apoptotic cell death [126]. A low level of lysosomal membrane permeabilization triggers apoptotic death, whereas a high level leads to rapid and direct cell death, resembling a form of necrosis.

b. Oleacein

Oleacein demonstrated an increase in internucleosomal DNA fragmentation in melanoma cells, indicating proapoptotic effects. This effect was accompanied by an elevation in proapoptotic BAX mRNA levels and a significant reduction in the expression of antiapoptotic proteins BCL-2 and MCL-1 genes. Interestingly, the modulation of pro and antiapoptotic gene expression occurred through the regulation of miRNAs capable of post-transcriptionally regulating gene expression. This regulation included a significant decrease in miR-214-3p, which targets BAX, as well as a significant upregulation of miR-34a-5p and miR-16-5p, both targeting BCL2, and miR-193a-3p, targeting MCL-1 [110]. In endothelial cells, oleacein notably increased the number of cells showing fragmented DNA (subG1 population), a characteristic event linked with the induction of apoptosis, followed by the activation of caspase-7 and -3 [109]. Oleacein induces apoptosis in neuroblastoma cells via upregulating BAX and downregulating BCL-2 protein expression [111].

c. Oleuropein

Oleuropein induces apoptosis in several cancer cells via activation of an intrinsic mitochondrial pathway through upregulation of a pro-apoptotic Bax protein while the levels of antiapoptotic proteins such as BCL2 and survivin decrease leading to the activation of apoptosis machinery [48,60,63,71,76,80,85,91]. Seçme et al. indicated that the mRNA expression of p53 increases in cells treated with oleuropein. Additionally, the mRNA expression of proapoptotic genes Bax, Bid, and Bad is elevated, while the mRNA expression of the antiapoptotic gene Bcl-2 is decreased [76]. In MCF7 cells, oleuropein induces cell cycle arrest at the G1 phase, which aligns with previous findings [51]. The increase in the sub-G1 phase after oleuropein treatment was also observed in lung cancer cells (A549 and H1299) followed by increased cleaved-PARP protein [80,85]. Oleuropein is also linked to another apoptosis inducer, Glyoxalase 2 (Glo2), an ancient enzyme categorized within the glyoxalase system, which demonstrates significant proapoptotic effects [127]. Antognelli et al. reported that oleuropein induces apoptosis in NSCLC cells by upregulating mGlo2, mediated by the superoxide anion and the Akt signalling pathway. Furthermore, they demonstrated that the proapoptotic effect of mGlo2 is due to the interaction of mGlo2 with the proapoptotic Bax protein [89].

Oleuropein induces autophagy in neuroblastoma cells through the Ca^2+^-CAMKKβ–AMPK axis. This leads to a swift release of Ca^2+^ from the SR stores, subsequently activating CAMKKβ. This activation results in the phosphorylation and activation of AMPK, leading to the inhibition of phospho-mTOR immunoreactivity and reduced levels of phosphorylated mTOR substrate p70 S6K [62]. In triple-negative breast cancer, oleuropein inhibits cell migration and invasion induced by HGF through autophagy by upregulation of LC3-II/LC3-I and Beclin-1, as well as downregulation of p62 [68].

#### 3.1.3. Inhibition of Angiogenesis

Angiogenesis, which involves the creation of new blood vessels from pre-existing vasculature, plays a crucial role in the later phases of carcinogenesis. It enables tumors to expand beyond a diameter of 1–2 mm, infiltrate surrounding tissues, and ultimately metastasize [128]. Numerous chemopreventive agents, both natural and synthetic, currently under development or in clinical use, demonstrate the inhibition of new blood vessel formation.

- Oleocanthal

Oleocanthal hinders vascularization in the chorioallantoic membrane [129] of fertilized chicken eggs, exerting an inhibitory effect on angiogenesis [109]. Additionally, oleocanthal decreases viability, migratory capacity, invasive potential, and the formation of tubular-like structures of endothelial cells and human umbilical vascular endothelial cells (HUVEC) [40,44,109]. HUVECs are commonly employed as a model system for investigating the regulation and development of angiogenesis. Moreover, at the molecular level, this inhibitory effect is likely mediated through the suppression of the phosphorylation of AKT, ERK1/2 [109], and c-Met kinase [40].

b. Oleacein

Oleacein inhibits a crucial step in angiogenesis, activating proliferation in quiescent endothelial cells, which supports blood vessel formation. Additionally, oleacein suppresses the migratory and invasive capabilities of endothelial cells and the formation of tubular-like structures by these cells. Moreover, in vivo CAM assays demonstrate that treatment with oleacein leads to reduced and impaired vascularization beneath the disc and surrounding area [109].

c. Oleuropein

Oleuropein disrupts the formation of melanoma tubes by inducing cell rounding and inhibiting tube retraction. Furthermore, it disrupts the organization of actin filaments within cells, thereby affecting the cytoskeleton [52]. Additionally, in vivo studies have revealed that oleuropein inhibits the high-fat diet-induced accumulation of adipocytes and M2-MΦs, as well as the expression of VEGF-A, -D, and HIF-1α in tumor tissues. Consequently, this suppresses tumor angiogenesis and lymphangiogenesis in melanoma-bearing obese mice [103]. The senescent fibroblast’s SASP generates a vasculogenic environment that promotes the formation of vessels by endothelial progenitor cells [130]. Oleuropein inhibits the tube formation and migration of endothelial cells, which are dependent on fibroblast SASP, by modulating the secretion of pro-angiogenic factors in the cellular microenvironment such as MMP-2, MMP-9, and uPA [101].

#### 3.1.4. Inhibition of Metastasis

The term “metastasis” describes the migration of cancer cells from the original tumor to adjacent tissues and distant organs, constituting the foremost factor contributing to cancer-related morbidity and mortality [131]. During the early stages of metastasis, cancer cells penetrate the basement membrane and traverse the tumor stroma [132]. In this step, the epithelial to mesenchymal transition (EMT) becomes a crucial factor. EMT is a biological process in which epithelial cells transform, leading to the loss of their cell polarity and cell–cell adhesion. These cells acquire invasive and migratory characteristics, transforming into mesenchymal stem cells [133].

- Oleocanthal

Oleocanthal suppresses epithelial-to-mesenchymal transition in human hepatocellular carcinoma and breast cancer [33,34]. It has been observed that oleocanthal enhances the expression of the epithelial marker E-cadherin while simultaneously reducing the expression of mesenchymal markers N-cadherin and vimentin. This modulation of proteins associated with epithelial–mesenchymal transition (EMT) occurs through the downregulation of twist transcription factors. The downregulation is achieved by diminishing the binding between STAT3 and the twist gene promoter, thereby inhibiting the activity of the twist promoter. Moreover, oleocanthal demonstrates the inhibition of IL6-induced activation of STAT3 in HEPG2 cells [33]. In an in vivo investigation using breast cancer orthotopic xenograft tumors, the administration of oleocanthal exhibited the capacity to stabilize E-cadherin while concurrently reducing vimentin expression in recurrent tumors derived from both BT-474 and MDA-MB-231. Additionally, oleocanthal also resulted in a decrease in the phosphorylation levels of both MET and HER2 in recurrent tumors originating from animals inoculated with BT-474 [34]. Hepatocyte growth factor (HGF) is recognized for its role in promoting the development and progression, including metastasis, of breast carcinoma [134]. HGF stimulates cell migration in breast cancer cells and oleocanthal attenuates this effect by suppressing the phosphorylation of Brk, paxillin, Rac1, and c-Met. This suggests that inhibition, at least in part, occurs through the suppression of the Brk/paxillin/Rac1 and the c-Met signalling pathway [39].

b. Oleacein

One of the key characteristics of metastatic cancer cells is their capacity to adhere to extracellular matrices. Notably, oleacein effectively suppressed the adhesion of neuroblastoma cells to collagen I-coated wells. Furthermore, oleacein also hindered cell migration in these models [111].

c. Oleuropein

Oleuropein inhibits breast cancer cells migration and invasion ability [52]. In MCF-7 breast cancer cells, oleuropein inhibits the migration ability of cells by suppressing EMT through increasing the expression of p53, which increases miR43a expression that leads to the downregulation of sirtuin-1 (SIRT1), resulting in the downregulation of mesenchymal markers (for example, matrix metalloproteinase-2 and -9 (MMP-2 and MMP-9)), downregulation of the EMT inducer (ZEB1), and upregulation of the epithelial marker (E-CAD) [92,135]. Interestingly, it has been reported that SIRT1 forms a complex with ZEB1 at the E-CAD promoter, deacetylating histones H4K16 and H3K9, which reduces RNA Pol II binding to the E-CAD promoter and subsequently suppresses its transcription [136]. Interestingly, oleuropein in N2a cells downregulated p53 and upregulated SIRT1 [137]. The difference is caused by the presence of estrogen receptors: in MCF-7 cells, the oleuropein bonds to the mentioned receptors, while estrogen receptors in N2a cells are missing [138]. 

Moreover, oleuropein reduces invasiveness of MCF-7 cells via dose-dependently reducing the expression of the epigenetic factor HDAC4 [82]. Additionally, in triple-negative breast cancer cells (TNBCs), oleuropein modulates the miR-194/XIST/PD-L1 loop by decreasing PD-L1 and miR-194 levels while upregulating XIST, thereby contributing to its ability to reduce the migration capacity of MDA-MB-231 cells [75].

#### 3.1.5. Modulation of Cancer-Associated Signaling Pathways

Ever since the initial identification of oncogenes such as BRAF, MYC, KIT, and RAS, along with tumor suppressor genes like BRCA1, TP53, and PTEN, genetic abnormalities associated with cancer have been extensively documented [139]. Presently, signalling pathways and molecular networks are acknowledged for their crucial roles in governing vital pro-survival and pro-growth cellular processes. Consequently, they are primarily implicated not only in the initiation of cancer but also in its potential treatment [140]. The PI3K/AKT/mTOR and RAF/MEK/ERK pathways commonly undergo activation and dysregulation across nearly all types of neoplasms, often exhibiting alterations in their various components. These RAF/MEK/ERK signalling cascades facilitate the transmission of signals from the cell surface to the nucleus, thereby modulating gene expression, cell cycle progression, and apoptosis [141,142].

- Oleocanthal

Oleocanthal inhibits the ERK1/2 and AKT oncogenic pathways in both melanoma and endothelial cells [28,109]. This inhibition leads to the downregulation of the antiapoptotic protein BCL-2, consequently reducing the viability of melanoma cells [28]. Furthermore, it demonstrates an antiangiogenic effect by suppressing endothelial cell proliferation, invasion, and tube formation [109]. According to molecular modelling studies, it has been suggested that oleocanthal demonstrates nine out of ten crucial binding interactions akin to those observed with a potent natural inhibitor of dual PIK3-γ/mTOR. Remarkably, the administration of oleocanthal resulted in a notable reduction in phosphorylated mTOR within the metastatic breast cancer cell line (MDA-MB-231) [37]. Interestingly, oleocanthal inhibits the upstream signalling of mTOR and MAPK, particularly targeting SMYD2, which plays a crucial role in prostate cancer proliferation and recurrence [27]. Another signalling pathway playing a significant role in cancer progression is STAT-3, which regulates numerous cellular processes such as the cell cycle, cell proliferation, cellular apoptosis, and tumorigenesis. Persistent activation of STAT-3 has been observed in various cancer types, and a high level of phosphorylation of STAT-3 may be linked to a poor prognosis in cancer [143]. Oleocanthal inhibits the phosphorylation and nuclear translocation of STAT3 in melanoma [44] and hepatocellular carcinoma [33]. Oleocanthal has also demonstrated the ability to inhibit the Brk/Paxillin/Rac1 pathway by suppressing Brk phosphorylation, subsequently leading to the downregulation of Rac1 and paxillin phosphorylation. These inhibitory effects have contributed significantly to the suppression of migration and invasion abilities in HGF-induced breast cancer cells [39].

b. Oleacein

Oleacein in melanoma cells reduces the mRNA expression of c-KIT, K-RAS, and PIK3R3, which are crucial effectors responsible for heightened mTOR activation. When hyperactivated, mTOR signalling facilitates cell proliferation and metabolism, which contribute to both tumor initiation and progression [144]. Interestingly, oleacein counters the expression of these genes by inducing an increase in the expression of miR-34a-5p, miR-193a-3p, miR-193a-5p, miR-16-5p, and miR-155-5p [110]. These microRNAs serve as regulatory elements capable of decreasing the transcriptional levels of the target genes (c-KIT, K-RAS, and PIK3R3) [145,146,147]. In endothelial cells, oleacein hampers the PI3K/AKT and ERK/MAPK signalling pathways, which may contribute to its antiangiogenic effect [109]. In neuroblastoma cells, oleacein decreases the phosphorylation of STAT3, which may be associated with the observed increasing expression level of Bax and P53 genes as well as the decrease in Bcl-2, ultimately leading to apoptosis induction in these cells [111].

c. Oleuropein

The NFκB signalling pathway plays a central role in the development and progression of cancer, exerting extensive involvement. NFκB facilitates tumor cell survival, proliferation, and angiogenesis by regulating the expression of target genes such as cyclin D1, IL6, BCLXL, BCL2, XIAP, and VEGF [148]. In breast cancer cells, oleuropein downregulates NFκB and concurrently suppresses cyclin D1, which is one of the most important targets of NFκB [48].

Another crucial signalling pathway in cancer is the MAPKs. The involvement of mitogen-activated protein kinases (MAPKs) in the initiation and progression of cancer have become more and more recognized. They have critical roles in cell differentiation, proliferation, and apoptosis. The MAPK family comprises extracellular signal-regulated kinase p38 MAPK, (ERK p44/42) and c-Jun N-terminal kinase (JNK) [149,150]. In lung cancer, the A549 and H1299 cells exhibited an increase in the phosphorylation of the p38MAPK protein in response to oleuropein, but oleuropein did not activate JNK [80,85]. Subsequently, this resulted in the activation of the ATF-2 via phosphorylation, which is a downstream factor of p38MAPK. Notably, in the cells treated with oleuropein, activation of the p38MAPK pathway induced apoptosis in a dose-dependent manner, with the maximum concentration of 200 μM [80,85]. Additionally, oleuropein inhibits the E2-dependent activation of ERK1/2 in breast cancer [54] and thyroid cancer cells [78].

The PI3K/AKT/mTOR (PAM) signalling pathway constitutes a profoundly conserved signal transduction network within eukaryotic cells, fostering cell survival, growth, and progression through the cell cycle. Dysfunctions within this pathway, such as PI3K hyperactivity, PTEN loss of function, and AKT gain of function, are notorious catalysts of treatment resistance and cancer progression [129]. In glioma cells, oleuropein attenuates AKT signalling, leading to the upregulation of BAX and the downregulation of BCL-2, resulting in reduced cell viability and induction of apoptosis [88]. Oleuropein in a concentration of 100 μM and 500 μM in a dose-dependent manner also suppresses AKT signalling by inhibiting pAkt (Ser473) and Akt (Thr308) in LNcaP and DU145 prostate cancer cell lines and BPH-1 non-malignant cells; however, in the DU145 cells, AKT activity is generally higher. Furthermore, the oleuropein increased the intracellular quantity of the protective thiol group and reduced the ROS activity in the examined cell lines, but in DU145 cells, the oleuropein in 500 μM concentration paradoxically decreased thiol group quantity and increased the measured ROS activity [61]. Moreover, oleuropein hinders mTOR signalling pathways and induces autophagy in TgCRND8 mice [62].

#### 3.1.6. Disruption of Redox Hemostasis and Endoplasmic Reticulum Stress

An imbalance between the production of reactive oxygen species (ROS) and the cell’s natural antioxidant defenses leads to oxidative stress, which has been connected to the emergence of cancer. ROS disrupts redox homeostasis, leading to the initiation of abnormal signalling networks that foster tumorigenesis and promote tumor formation [151]. Reactive oxygen species (ROS), such as superoxide radicals (O_2_^−^), hydrogen peroxide (H_2_O_2_), and hydroxyl radicals (OH), are chemically active molecules that play a crucial role in the biological functions of tumor cells. Reactive oxygen species (ROS) demonstrate paradoxical effects on cancers. At mild or even in low concentration, they promote tumorigenesis and facilitate the progression of cancer cells. Conversely, an excessive concentration results in cell death but depends on the source and type of ROS, too [152].

- Oleocanthal

The administration of oleocanthal induced the expression of γH2AX, a marker indicating DNA damage, and provoked a dose-dependent increase in intracellular ROS production. Additionally, it led to mitochondrial depolarization, resulting in the inhibition of colony formation capacity in hepatocellular carcinoma and colorectal carcinoma cells [32]. In another study, oleocanthal was found neurotoxic through oxidative stress in neuroblastoma cells by decreasing their viability, while in BMDN cells, the cytotoxicity was negligible. Notably, there was a significant rise in neuroblastoma cells in the immunoreactivity of i-NOS and e-NOS, which serve as indicators for evaluating oxidative stress [43]. The generation of reactive oxygen species (ROS) observed triggers the opening of the mitochondrial permeability transition pore. This, combined with the swift cleavage and activation of initiator caspases, constitutes pivotal components of the intrinsic apoptosis pathway [153].

b. Oleuropein

Oleuropein exhibits beneficial effects as an anticancer agent in the N-ethyl-N-nitrosourea (ENU)-induced brain tumor model in Wistar rats through redox control mechanisms involving endogenous non-enzymatic and enzymatic antioxidant defense systems. These effects are dependent on the gender of the animals: namely the gene expression and activity of catalase enzymes were increased in male rats [66]. Jamshed et al. reported that oleuropein possesses potent free radical scavenging properties and effectively inhibits the formation of 8-hydroxydeoxyguanosine (8-OH-dG) in tamoxifen-induced oxidative DNA damage in Balb/c mice [105]. In thyroid cancer cells, oleuropein has the capability to decrease the proliferation, and this effect is linked to a reduction in H_2_O_2_-induced ROS levels [78]. Interestingly, at elevated doses, oleuropein impedes the growth and viability of ovarian cancer cells by increasing the production of reactive oxygen species (ROS) and levels of LIP. Consequently, this disrupts the S-phase of the cell cycle and initiates apoptosis [90].

## 4. Discussion

The well-documented health benefits associated with the Mediterranean diet, with extra virgin olive oil as a cornerstone, are strongly supported by extensive epidemiological research. Secoiridoids from *O. europaea* such as oleocanthal, oleuropein, and oleacein exhibit broad therapeutic potential, adeptly addressing a variety of health concerns ranging from inflammatory and neurodegenerative conditions to cancer. To the best of our knowledge, this systematic review provides the first comprehensive summary of the chemopreventive potential of oleocanthal, oleuropein, and oleacein. Our findings not only corroborate the chemopreventive efficacy of these compounds but also reveal new insights into their mechanism of action across different cancer cell lines. Compared to the previous review by Fabiani. [4], which focused primarily on the in vivo anti-cancer activities of secoiridoid phenols, and the narrative review by Emma et al. [5], which provided a critical analysis of preclinical studies, our review offers a detailed mechanistic analysis and includes the latest studies up to 10 October 2023. This review highlights the pleiotropic effects of secoiridoids across different tissue types, emphasizes their role in modulating various pathways involved in cancer progression, and comprehensively combines in vitro and in vivo studies examining the use of these compounds to treat inflammation and various types of cancer. Also, in our systematic review, we complement the narrative review of Emma et al. about the preclinical effects of the most relevant secoiridoids by providing novel insights into the specific mechanisms of action and detailed chemopreventive effects of oleocanthal, oleacein, and oleuropein across different cancer cell lines.

The three compounds are chemically related, and although their mechanisms of action are somehow different, the key elements are overlapping. The ability to inhibit cell proliferation and induce cell cycle arrest is essential, addressing one of the hallmarks of cancer cells: the capacity to grow continuously even in the absence of external stimuli. Moreover, the role of secoiridoids in inducing apoptosis offers a direct mechanism to increase the survival of patients with cancer. Apoptosis is a natural barrier to cancer progression, which is often circumvented or suppressed in tumor cells. The anti-angiogenic and anti-metastatic activities of these compounds further expand their therapeutic potential. By inhibiting angiogenesis, secoiridoids prevent tumors from developing new blood vessels needed for oxygen and nutrient supply, thus effectively starving the tumor and limiting its growth and spread. Similarly, by suppressing epithelial to mesenchymal transition (EMT), oleocanthal and related compounds inhibit the ability of cancer cells to invade surrounding tissues and metastasize to distant organs, a critical step in the progression of cancer. Moreover, their impact on redox homeostasis and induction of oxidative stress in cancer cells taps into the balance between ROS production and antioxidant defense, driving cancer cells towards apoptosis through oxidative damage without harming the normal cells. For the sake of completeness, we need to mention that it is virtually contradicting that oleuropein in breast cancer cells decreases the SIRT1 level and in gastric adenocarcinoma cells it increases the KRAS while oleacein in 501Mel cells decreases the KRAS and mTOR expression but all mechanisms lead to chemopreventive effects [91,92,109]. This is explained by the fact that secondary signal transducers correlated to tumor suppressors (such as SIRT1) or potential oncogens (for example, mTOR, and KRAS) behave pleiotropically depending on the tissue or cell type. For example, both SIRT1 and P53 contribute to the effects of chemopreventive agents if examined in a stand-alone experiment. Practically, their balanced activity contributes to maintaining cell homeostasis regarding proliferation [154]. Increasing p53 gene expression generally exerts tumorsuppressor effects, while the same is true on SIRT1 through its mTOR and NFκB inhibiting effect, but SIRT1 and p53 can cross-regulate each other [92,155], namely the deacethylase activity of SIRT1 can deactivate p53. Hence, oleuropein exerts pleiotropic effects on intracellular signal transduction, namely that estrogen receptor is needed for oleuropein to increase p53 activity. 

Both olive oil and olive leaf are chemically complex matrices that comprise a variety of chemical substances. Secoiridoids are important in the effects of these plant compounds, reducing the risk of various chronic diseases including cancer, even though they are not the most important constituents quantitatively. Their role in the chemopreventive effects of olive oil and leaf is unquestionable, and the mechanisms of their effects are confirmed in several experiments, as summarized in our review. The synergies of the mentioned chemopreventive mechanisms are obvious in smaller doses, too, since the in vitro literature mentions 500 μM concentrations of oleuropein as maximal effective doses. To reach such a quantity in vivo, however, consumption of approximately 35 kg of extra virgin olive oil is required. On the other hand, a 20 µmol concentration of oleocanthal was also mentioned as an effective in vivo dose.

This review highlights the distinct anticancer mechanisms of oleocanthal, oleacein, and oleuropein, despite their shared structural features. Oleocanthal primarily induces apoptosis through lysosomal membrane permeabilization, leading to cellular toxicity and reduced tumor burden. Oleacein, on the other hand, is notable for its anti-angiogenic and anti-metastatic effects, which prevent tumor spread and the formation of new blood vessels. Oleuropein exhibits a more variable profile; it can either decrease levels of tumor suppressors like SIRT1 or alter oncogene expressions such as KRAS and mTOR, depending on the cancer type. While all three compounds share common mechanisms, such as cell cycle arrest and modulation of redox homeostasis, their individual pathways contribute uniquely to their chemopreventive activities. This detailed comparison underscores the diverse mechanisms through which these secoiridoids exert their anticancer effects, enhancing their potential as therapeutic agents in cancer treatment.

## 5. Conclusions

In summary, the beneficial effects of secoiridoid intake, for example, in an increased dose utilizing food supplements is underpined. The clinical significance of the chemopreventive effects is supported by the negative correlation between olive oil consumption and cancer risk according to epidemiological studies. Compared to their counterparts, oleacein was the least explored and was only studied in four articles. Notably, none of these studies reported its activity in disrupting redox hemostasis and endoplasmic reticulum stress. Consequently, further investigations are warranted to unravel additional chemopreventive mechanisms attributed to oleacein. Moreover, given the documented chemopreventive properties of oleocanthal, oleuropein, and oleacein, it becomes imperative to substantiate their efficacy through robust randomized clinical trials. Such trials are essential to provide concrete clinical evidence supporting the incorporation of these compounds into mainstream clinical practices, thereby adding treatment strategies to prevent cancer or improve the life quality of patients with cancer by hindering progradiation and supporting chemoterapeutical medicines.

## Figures and Tables

**Table 1:** The chemopreventive effects of oleocanthal, oleuropein, and oleacein on targeted cancer cells (in vitro, in silico, and in vivo studies).

Compound	Study Design	Tissue/Cell Type	Major Finding	Mechanism	Ref.
**Oleocanthal**	in vitro and in vivo (mouse, RIP-Tag)	Pancreatic tumor/PC3, MDA-MB-231, MCF7, HEK-293T, MCF10A, BJ-hTert and PNET N134	Oleocanthal induces damage to cancer cell lysosomes, causing lysosomal membrane permeabilization and the release of cathepsin into the cytosol. This process leads to cellular toxicity, resulting in the inhibition of cancer cell viability, reduced tumor burden, and an extended lifespan observed in mice with pancreatic neuroendocrine tumors.	Inducing cell death (necrosis)	Goren et al. [26]
**Oleocanthal**	in vitro and in vivo (mouse, athymic nude, male)	Metastatic castration-resistant prostate cancer (mCRPC) tumor/DU-145, PC-3, CWR-R1ca and PC-3M	Oleocanthal suppresses the viability, migration, invasion, and colony formation of prostate cancer cell lines. It also reduces the progression and metastasis of mCRPC tumors in nude mice by suppressing the downstream substrates and signalling of SMYD2 (EZH2, p65, mTOR, MAPK), which are crucial for prostate cancer proliferation and relapse.	Inhibition of cell proliferation, metastasis, and modulation of cancer cell signalling pathways	Siddique et al. [27]
**Oleocanthal**	in vitro	A375, 501Mel and HDFa	Oleocanthal suppresses the viability of melanoma cells (A375 and 501Mel) by inhibiting the oncogenic pathways (ERK1/2 and AKT), resulting in the downregulation of the anti-apoptotic protein Bcl-2.	Induction of cell death (apoptosis) and modulation of cancer cell signalling pathways	Fogli et al. [28]
**Oleocanthal**	in vitro	MDA-MB-231, MCF7, G361, HT144, HCT 116, HeLa, MIA PaCa-2, A549, 293T, Jurkat, CEM, Raji, K-562, Caco-2, JEG-3 and NCI-H460	Oleocanthal suppresses the viability of cancer cells and induces apoptotic cell death via activation of the intrinsic apoptotic pathway (ROS production and mitochondrial membrane depolarization).	Inducing cell death (apoptosis)	Pastorio et al. [29]
**Oleocanthal**	in vitro, in silico and in vivo (mouse, athymic nude, female)	BT-474 tumor xenografts/BT-474, MCF-7, and T-47D	Oleocanthal, both alone and in combination with tamoxifen, inhibits the growth of luminal breast cancer cells. Additionally, oleocanthal reduces the growth of the BT-474 tumor. These effects are partly attributed to the reduction in total Erα levels in both cell culture and animal studies. Based on in silico studies, oleocanthal is hypothesized to directly interact with the Erα receptor, thereby modulating the pharmacological signalling of this crucial nuclear hormone receptor.	Inhibition of cell proliferation	Ayoub et al. [30]
**Oleocanthal**	in vitro	MCF10A, MCF7 and MDA-MB-231	Oleocanthal disrupts the ability of breast cancer cells to proliferate and migrate through the downregulation of TRPC6 channel expression. Additionally, selective activation of TRPC6-dependent Ca^2+^ influx leads to Ca^2+^ entry and mobilization.	Inhibition of cell proliferation and inhibition of cell migration	Diez-Bello et al. [31]
**Oleocanthal**	in vitro	HepG2, Hep3B, Huh7, PLC/PRF/5, SW480 and HT29	Oleocanthal suppresses colony formation ability and induces apoptosis in colorectal and hepatocellular carcinoma cells through the upregulation of reactive oxygen species (ROS).	Induction of cell death (apoptosis) and disruption of redox hemostasis	Cusimano et al. [32]
**Oleocanthal**	in vitro and in vivo (mouse, athymic nude, male)	Orthotopic tumor model of hepatocellular carcinoma (HCC)/LO2, HCCLM3, HepG2 and Huh-7	Oleocanthal inhibits proliferation, induces apoptosis, and suppresses cell migration in HCC cells. Moreover, it suppresses tumor growth and metastasis in an orthotopic HCC model. These effects are attributed to the reduction in STAT3 nuclear translocation and DNA binding activity, downregulating its downstream effectors (cyclin D1, Bcl-2 survivin, MMP-2).	Induction of cell death (apoptosis), inhibition of metastasis, inhibition of cell proliferation, and modulation of cancer cell signalling pathways	Pei et al. [33]
**Oleocanthal**	in vivo (mouse, athymic nude, female)	BT-474 and tumor xenografts	Oleocanthal reduces the growth of recurrent breast cancer tumors. It upregulates the expression of the epithelial marker E-cadherin, downregulates the levels of the mesenchymal marker vimentin, and also reduces the activation of MET and HER2 receptors.	Inhibition of cell proliferation and metastasis	Siddique et al. [34]
**Oleocanthal**	in vitro	BxPC3, PC3, MDA-MB-231, BJ, 3Y1, and IMR90	Oleocanthal triggers both apoptotic and necrotic cell death by inducing lysosomal membrane permeabilization (LMP). This occurs through the inhibition of acid sphingomyelinase [35] activity, leading to the destabilization of the interaction between proteins crucial for maintaining lysosomal membrane stability.	Inducing cell death (apoptosis and necrosis)	LeGendre et al. [36]
**Oleocanthal**	in vitro and in silico	MCF-7, T47D, Caco-2, MDA-MB-231 and HeLa	Oleocanthal reduces the viability of cancer cells. Molecular modeling studies have indicated that oleocanthal exhibits nine out of ten critical binding interactions similar to those of a potent dual PIK3-γ/mTOR natural inhibitor. Notably, treatment with oleocanthal significantly decreases the level of phosphorylated mTOR in the metastatic breast cancer cell line (MDA-MB-231).	Inhibition of cell viability	Khanfar et al. [37]
**Oleocanthal**	in vitro and in vivo (athmyc nude mouse, Foxn1nu/Fox+, female)	A549-Luc LC Metastasis/A549, A549-Luc and NCI-H322M	Oleocanthal exerts inhibitory effects on HGF-mediated proliferation and migration in human LC cell lines A549 and NCI-H322M cells. These effects are achieved through the dual targeting of c-MET and COX-2. The study demonstrated a significant decrease in total and activated c-MET levels and the inhibition of COX1/2 activity in lung adenocarcinoma cells. Additionally, oleocanthal treatments led to a notable inhibition of the progression and metastasis of NSCLC A549-Luc cells in a nude mouse tail vein model. c-MET activation was reduced in tumor cell lysates. Moreover, microarray data from oleocanthal-treated lung tumors revealed a distinct gene signature confirming the dual targeting of COX2 and c-MET.	Inhibition of cell proliferation and metastasis	Siddique et al. [38]
**Oleocanthal**	in vitro and in vivo (athymic nude mouse, Foxn1nu/Fox+, female)	MDA-MB-231/GFP-breast tumor/MDA-MB-231, MDA-MB-231/GFP, MCF-7, MCF10A and BT-474	Oleocanthal inhibits the viability of various human breast cancer cell lines, namely MDA-MB-231, MCF-7, and BT-474, with a pronounced impact on apoptosis, particularly in MDA-MB-231 cells. Moreover, oleocanthal exhibits a dose-dependent suppression of HGF-induced cell migration, invasion, and G1/S cell cycle progression in MDA-MB-231 cells. Notably, the effects of oleocanthal are determined to be mediated through the inhibition of Brk/paxillin/Rac1 signalling pathways and the suppression of HGF-induced c-Met activation, along with subsequent downstream mitogenic signalling pathways. Additionally, oleocanthal plays a role in stabilizing the epithelial phenotype, as evidenced by increased expression of E-cadherin and zona occludens 1, while concurrently reducing the mesenchymal phenotype, indicated by decreased vimentin expression in breast cancer cells.	Inhibition of cell proliferation, induction of cell death (apoptosis), inhibition of metastasis and modulation of cancer cell signalling pathways	Akl et al. [39]
**Oleocanthal**	in vitro and in silico	MCF7, MDA-MB-231 and prostate cancer cell line PC-3	Oleocanthal inhibits the proliferation, migration, and invasion of epithelial human breast and prostate cancer cell lines. Additionally, it exhibits anti-angiogenic activity by suppressing the expression of the microvessel density marker CD31 in endothelial colony-forming cells.	Inhibition of cell proliferation, inhibition of cell migration, and inhibition of angiogenesis	Elnagar et al. [40]
**Oleocanthal**	in vivo (transgenic mouse, FVB/N-Tg (MMTV-P), female)	Lung of Transgenic MMTV-PyVT Mice and Breast Cancer Patient-Derived Xenograft (PDX) Model	Oleocanthal inhibits the growth and metastasis of breast cancer. According to the ingenuity pathway analysis, there is a notable overlap in the top-downregulated pathways, including the PI3K-AKT, PD-1/PD-L1, and protein kinase A signalling pathways, in PDX and MMTV-PyVT mouse tumors after oleocanthal treatment. This implies a potential suppression effect on non-melanoma, glioma, colorectal, lung, breast, and prostate cancers.	Inhibition of cell proliferation and metastasis	Qusa et al. [41]
**Oleocanthal**	in vitro and in vivo (mouse, Swiss albino, male and female)	BT-474 BC cell xenograft model/MDA-MB-231 and BT-474	Oleocanthal formulation EF-2 inhibits locoregional recurrence in BT-474 tumor cells following surgical excision of the primary tumor. Moreover, it suppresses over 70% of both hormone and HER2-positive BT-474 breast cancer cell growth in a nude mouse xenograft model.	Inhibition of proliferation	Tajmim et al. [42]
**Oleocanthal**	in vitro	NB2a and BMSCs	Oleocanthal suppresses proliferation of neuroblastoma cells by inducing apoptosis and oxidative stress through the upregulation of i-NOS and e-NOS.	Inhibition of proliferation, inducing cell death (apoptosis) and disruption of redox hemostasis	Ünsal et al. [43]
**Oleocanthal**	in vitro	A375, A2058, HUVEC and HaCaT	Oleocanthal inhibits the proliferation, angiogenesis, and metastasis of cancer cells by inhibiting the phosphorylation and nuclear translocation of STAT3. This inhibition results in the downregulation of STAT3 target genes, such as Bcl-xL, Mcl-1, MMP-2, VEGF, and MMP-9. These genes play crucial roles in the processes of apoptosis, invasion, and angiogenesis in melanoma.	Inhibition of angiogenesis, inducing cell death (apoptosis), inhibition of metastasis and modulation of cancer cell signalling pathways	Gu et al. [44]
**Oleuropein and oleocanthal**	in vitro	MDA-MB-231 and MDA-MB-468	Oleuropein and oleocanthal exhibit anti-proliferative effects in triple-negative breast cancer (TNBC) cell lines by impeding cell survival and modifying the gene expression of these cells. Oleocanthal demonstrates a more potent inhibition of cancer cells’ viability compared to oleuropein. Pathway analysis unveiled several pathways linked to TNBC, including apoptotic processes, cell death, cellular response to stress, inducing cell cycle arrest by p21, inhibiting cancer progression, and invasion through decreasing MMPs expression.	Inhibition of cell proliferation	Karousi et al. [45]
**Oleuropein**	in vitro	SEM-1, HepG2 and TCAM-2	Oleuropein reduces the viability of seminoma cells by inducing apoptosis through the inhibition of NF-κB nuclear translocation, resulting in the inhibition of cyclin-D1 expression and the upregulation of p21^Cip/WAF1^. Additionally, oleuropein suppresses the cellular motility and migration of seminoma cells by downregulating TGFβ-1 expression.	Inducing apoptosis and inhibition of cell migration	Bossio et al. [46]
**Oleuropein**	in vitro	J774A.1 and mouse peritoneal macrophages	Oleuropein enhances the macrophage-mediated response, leading to increased production of nitric oxide (NO).	Antioxidative properties	Visioli et al. [47]
**Oleuropein**	in vitro	MCF-10A, MDA-MB-231 and MCF-7	Oleuropein reduces the viability of breast cancer cells by inducing apoptosis through modulation of mitochondrial pathway (↑ Bax, ↓ Bcl-2, ↓ survivin). Oleuropein also inhibits breast cancer cell proliferation by downregulating NF-kB and cyclin D1 but activates p21, leading to cell cycle arrest at the S phase.	Induction of cell death (apoptosis), inhibition of cell proliferation, and modulation of cancer cell signalling pathways	Elamin et al. [48]
**Oleuropein**	in vitro	143B OS	Oleuropein inhibits the proliferation and migration of highly metastatic osteosarcoma cells, and these effects are augmented when combined with 2-methoxyestradiol. Furthermore, oleuropein, whether administered alone or in conjunction with 2-methoxyestradiol, triggers autophagy in these highly metastatic osteosarcoma cells.	Inhibition of cell proliferation, autophagy, and inhibition of cell migration	Przychodzen et al. [49]
**Oleuropein**	in vitro	A375	Oleuropein inhibits cell proliferation and induces apoptosis by suppressing the pAKT/pS6 pathway. It enhances the cytotoxic impact of Dacarbazine (DTIC). Oleuropein has been observed to synergize with Everolimus (RAD001) and exhibit efficacy against PLX4032-resistant BRAF melanoma cells, suggesting potential collaboration in inhibiting the pAKT/pS6 pathway.	Inhibition of cell proliferation and induction of cell death (apoptosis)	Ruzzolini et al. [50]
**Oleuropein**	in vitro	MCF-7	Oleuropein induces apoptosis and suppresses cell proliferation by triggering G1 cell cycle arrest in MCF-7 cells.	Inhibition of cell proliferation and induction of cell death (apoptosis)	Han et al. [51]
**Oleuropein**	in vitro and in vivo (mouse, Swiss albino, male and female)	Soft tissue sarcomas/NL-Fib, LN-18, TF-1a, MCF-7, 786-O, T-47D, RPMI-7951, and LoVo	Oleuropein inhibits the growth and migration of various tumor cell lines. In a newly developed tube-disruption assay, oleuropein hampers angiogenesis by interrupting the division, motility, and invasiveness of cells through the disruption of their cytoskeleton, particularly the organization of actin filaments. When orally administered to mice with spontaneously developing tumors (soft tissue sarcomas), oleuropein effectively regresses tumor growth.	Inhibition of cell proliferation, inhibition of angiogenesis, and inhibition of cell migration	Hamdi et al. [52]
**Oleuropein**	in vitro	MG-63	Oleuropein inhibits the proliferation of osteosarcoma cells on its own, and when combined with adriamycin, it further enhances this inhibitory effect. Notably, oleuropein also increases the expression of autophagy-related genes (AMBRA1, ULK1, and BNiP3L). Additionally, the levels of LC3B are significantly reduced after treatment with oleuropein alone and in combination with adriamycin. Consistent with these findings, oleuropein markedly amplifies the expression of p62, an autophagy substrate.	Inhibition of cell proliferation and induction of autophagy	Gioti et al. [53]
**Oleuropein**	in vitro	MCF-7	Oleuropein inhibits the growth of estradiol-induced MCF-7 cells by modulating ERα transactivation, resulting in the inhibition of estradiol-dependent ERK1/2 activation.	Inhibition of cell proliferation and modulation of cancer cell signalling pathways	Sirianni et al. [54]
**Oleuropein**	in vitro	HUVECs and HMVECs-d-Ad	Oleuropein abrogates the tube formation of HUVECs cells.	Inhibition of angiogenesis	Lamy et al. [55]
**Oleuropein**	in vivo (mouse, albino hairless, male)	UVB irradiation induced skin tumor	Oleuropein demonstrates potent inhibition of skin carcinogenesis as well as the growth of blood vessels and tumors induced by UVB irradiation. Furthermore, it enhances the expression of MMP-2, MMP-9, and MMP-13, along with elevated levels of VEGF and COX-2 in the skin. Additionally, oleuropein impedes the increase in the expression of Ki-67 and CD31 in the skin of mice following UVB irradiation.	Inhibition of cell proliferation	Kimura et al. [56]
**Oleuropein**	in vitro	MCF-7 and MDA-MB-231	Oleuropein inhibits the viability, migration, and invasion of breast cancer cells while promoting apoptosis through the increased production of reactive oxygen species (ROS) and the inhibition of NF-κB activation in these cells.	Inhibition of cell proliferation, inhibition of cell migration, and modulation of cancer cell signalling pathways	Liu et al. [57]
**Oleuropein**	in vitro	HepG2, RKO and Huh7	Oleuropein inhibits the growth of HepG2 cells by triggering apoptosis, characterized by the upregulation of BAX and the downregulation of Bcl-2. This effect is achieved through the induced generation of reactive oxygen species (ROS), resulting in the inactivation of PI3K/AKT signalling.	Induction of cell death (apoptosis) and modulation of cancer cell signalling pathways	Yan et al. [58]
**Oleuropein**	in vitro and in silico	MDA-MB-231 and MCF-7 cells	Based on molecular docking analysis, oleuropein has been recognized as a potent molecule that forms a binding interaction with PAI-1. Additionally, oleuropein exhibits the capacity to act as a natural inhibitor of PAI-1 by progressively destabilizing PAI-1 levels, particularly in ER-/PR- breast cancer cells. This impact is accompanied by the suppression of cell growth and a downstream caspase activation.	Inhibition of cell proliferation	Tzekaki et al. [59]
**Oleuropein**	in vitro	MDA-MB-231 and MDA-MB-468	Oleuropein reduces cell viability by inducing cell cycle arrest and stimulating apoptosis in triple-negative breast cancer cells. In MDA-MB-468 cells, a significant increase in the expression of apoptosis-related genes is observed, particularly in two members of the caspase family (CASP1 and CASP14), proapoptotic genes (BNIP2, GADD45A, BID, BNIP3, and BCL10), and the TNF receptors TNFRSF21 and FADD. In MDA-MB-231 cells, RIPK2 and BIRC3 were upregulated and PYCARD and CASP6 were downregulated. Additionally, the antiapoptotic gene TNFRSF11B and the survivin BIRC5 were also downregulated.	Inhibition of cell proliferation and induction of cell death (apoptosis)	Messeha et al. [60]
**Oleuropein**	in vitro	BPH-1, LNCaP and DU145	Oleuropein reduces cell viability and induces changes in thiol groups, reactive oxygen species, γ-glutamylcysteine synthetase, and hemeoxygenase-1 in prostate cancer cell lines. Moreover, it suppresses Akt signalling by inhibiting pAkt(Ser473) and Akt(Thr308).	Inhibition of cell proliferation and modulation of cancer cell signalling pathways	Acquaviva et al. [61]
**Oleuropein**	in vitro and in vivo (mouse, TgCRND8)	TgCRND8 mice/SH-SY5Y and RIN-5F	Oleuropein induces autophagy by triggering the Ca^2+^-CAMKKβ–AMPK pathway. Additionally, Oleuropein inhibits mTOR through the activation of AMPK in the cortex of TgCRND8 mice. This suggests that the induction of autophagy by oleuropein develops through the AMPK/mTOR signalling pathway.	Induction of autophagy and modulation of cancer cell signalling pathways	Rigacci et al. [62]
**Oleuropein**	in vitro	MCF-7	Oleuropein induces apoptosis by upregulating the expression levels of both p53 and Bax genes while concurrently downregulating the expression of Bcl2.	Inducing cell death (apoptosis)	Hassan et al. [63]
**Oleuropein**	in vitro	MDA	Oleuropein prevents cancer metastasis by upregulating the TIMPs (TIMP1,-3, and -4) and downregulating the MMPs (MMP2 and MMP9) gene expressions.	Inhibition of metastasis	Hassan et al. [64]
**Oleuropein**	in vivo (mouse, C57BL/6, female)	AOM/DSS-induced CRC in C57BL/6 mice	Oleuropein inhibits the onset of colonic neoplasia in AOM/DSS-induced colorectal cancer (CRC) in mice by inhibiting inflammation in the colon and restricting the activation of STAT3, NF-κB, PI3K/Akt, and β-catenin.	Inhibition of cell proliferation	Giner et al. [65]
**Oleuropein**	in vivo (rat, Wistar, female)	N-ethyl-N-nitrosourea (ENU)-induced brain tumors	Oleuropein exhibits a restricted yet beneficial impact as an anticancer compound. These effects manifest through redox control mechanisms that engage both endogenous enzymatic and non-enzymatic antioxidant defense systems. Notably, the observed effects are closely tied to the gender of the animals.	Disruption of redox hemostasis	Ramírez-Expósito et al. [66]
**Oleuropein**	in vitro	MIA PaCa2, BxPC-3, CFPAC-1, HPDE and ASPC-1	Oleuropein arrests the cell cycle and induces apoptosis in pancreatic cancer cells by raising the Bax/Bcl-2 ratio. Moreover, oleuropein enhances the expression of c-Jun and c-Fos and thereby induces apoptosis.	Inducing cell death (apoptosis)	Goldsmith et al. [67]
**Oleuropein**	in vitro	MDA-MB-231	Oleuropein inhibits the viability of breast cancer cells and decreases cell migration and invasion through HGF or 3-MA induction causing autophagy. This is achieved by reversing the downregulation of LC3-II/LC3-I and Beclin-1, as well as the upregulation of p62.	Induction of authophagy	Lu et al. [68]
**Oleuropein**	in vitro	MCF-7 and MCF-10A	Oleuropein triggers apoptosis and significantly enhances the expression of Prdx genes in breast cancer cells.	Inducing cell death (apoptosis)	Junkins et al. [69]
**Oleuropein**	in vitro	MAT-LyLu	Oleuropein inhibits cell migration and suppress the invasiveness of MAT-LyLu cells. This effect was associated with the inhibition of VGSCs, primarily resulting from the direct reduction in mRNA expression of SCN9A.	Inhibition of cell migration	Aktas et al. [70]
**Oleuropein**	in vitro	MCF7 and MDA-MB-231	Oleuropein shows a substantial reduction in cell viability and induces apoptosis in breast cancer cells. Additionally, it elevates the expression levels of miR-125b, miR-16, miR-34a, p53, p21, and TNFRS10B while concurrently diminishing the expression of bcl-2, mcl1, miR-221, and miR-29a.	Inducing cell death (apoptosis)	Asgharzade et al. [71]
**Oleuropein**	in vitro	T98G	Oleuropein reduces cell viability and enhances the therapeutic effect of temozolomide in glioblastoma cells while also upregulating the expression of miR-181b, miR-137, and Let-7d in these cells.	Inhibition of proliferation	Tezcan et al. [72]
**Oleuropein**	in vitro	HT29	Oleuropein reduces cell viability, alters the distribution of the cell cycle, and triggers apoptosis, concurrently decreasing the expression of HIF-1α protein and elevating p53 protein levels in human colon cancer cells.	Inhibition of proliferation	Cárdeno et al. [73]
**Oleuropein**	in vitro	AGS	OLE reduces cell viability, causes DNA damage, and initiates apoptosis followed by necrosis in AGS cells. Furthermore, there is a significant decline in cell viability associated with elevated intracellular levels of reactive oxygen species (ROS).	Inhibition of proliferation	Türkdoğan et al. [74]
**Oleuropein**	in vitro	MDA-MB-231	Oleuropein inhibits the viability and migration capacity of breast cancer cells by decreasing the expression of miR194 and PD-L1 while concurrently upregulating the level of XIST.	Inhibition of proliferation and migration	Hamed et al. [75]
**Oleuropein**	in vitro	SH-SY5Y	Oleuropein induces apoptosis by activating the Bax gene and inhibiting the expression of Bcl-2. Additionally, it leads to cell cycle arrest by upregulating p53 and CDKN2A while simultaneously downregulating the expressions of CyclinD1, CyclinD2, CyclinD3, CDK4, and CDK6 genes.	Inhibition of proliferation and induction of cell death (apoptosis)	Seçme et al. [76]
**Oleuropein**	in vivo (A/J mouse, female)	AOM-induced colon tumorigenesis in A/J mice	Oleuropein inhibits the process of AOM-induced carcinogenesis in mice by diminishing the formation of dysplastic crypts in various segments of the colon. Additionally, it inhibits the progression from low dysplasia to high dysplasia. Moreover, OL has a capacity to decrease DNA damage in peripheral leukocytes.	Inhibition of proliferation	Sepporta et al. [77]
**Oleuropein**	in vitro	TPC-1 and BCPAP	Oleuropein inhibits the proliferation of thyroid cancer cells by suppressing the phosphorylation of ERK and Akt and decreasing levels of H_2_O_2_-induced reactive oxygen species (ROS).	Inhibition of proliferation, modulation of cancer cell signalling pathways, and disruption of redox hemostasis	Bulotta et al. [78]
**Oleuropein**	in vitro	MCF-7	Oleuropein reduces the proliferation of MCF-7 cells, enhances apoptosis, inhibits migration and invasion, and decreases the expression of miR-21 and miR-155.	Inhibition of proliferation and migration	Abtin et al. [79]
**Oleuropein**	in vitro	H1299	Oleuropein induces apoptosis and arrests the cell cycle at the G2/M phase. The mitochondrial pathway plays a crucial role in this process, involving an increase in the Bax/Bcl-2 ratio, release of cytochrome c, and activation of caspase-3. Additionally, the p38 MAPK signalling pathways are essential in the oleuropein-induced apoptosis.	Inhibition of proliferation, induction of cell death (apoptosis), and modulation of cancer cell signalling pathways	Wang et al. [80]
**Oleuropein**	in vitro and in silico	MCF-7	Oleuropein reduces the viability of breast cancer cells and triggers cell cycle arrest by reducing the activity of the phosphatase PTP1B.	Inhibition of proliferation	Przychodzen et al. [81]
**Oleuropein**	in vitro	MCF-7	Oleuropein inhibits the proliferation and invasion of cells by inducing apoptosis via the modulation of a crucial epigenetic factor, HDAC4.	Inhibition of proliferation and inhibition of cell invasion	Mansouri et al. [82]
**Oleuropein**	in vitro	HepG2	Oleuropein enhances the antitumor efficacy of cisplatin by modulating the regular proNGF/NGF equilibrium in HepG2 cells. This modulation is accomplished by modulating MMP-7 activity while leaving the gene expression of NGF unaffected.	Inhibition of proliferation	Sherif et al. [83]
**Oleuropein**	in vitro	A2780 S and A278/CP	Oleuropein inhibits cisplatin resistance in ovarian cancer by suppressing the expression of Bcl-2 and Mcl1 while concurrently enhancing the levels of P53, P21, and TNFRSF10B. Furthermore, there is a substantial decrease in the expression of miR-21 and an increase in the expression of miR-125b, miR-34a, and miR-16. These changes are potentially implicated in the reduction in cisplatin resistance.	Inhibition of proliferation	Sheikhshabani et al. [84]
**Oleuropein**	in vitro	A549 and BEAS–2 B	Oleuropein plays a role in causing G2/M phase cell cycle arrest and inducing apoptotic cell death in lung cancer cells. The activation of p38MAPK is crucial for oleuropein’s induction of apoptosis. Oleuropein triggers apoptosis by leading to a decrease in mitochondrial membrane potential, a decrease in Bcl-2 expression, and an increase in Bax expression.	Inhibition of proliferation, induction of cell death (apoptosis), and modulation of cancer cell signalling pathways	Cao et al. [85]
**Oleuropein**	in vitro and in vivo (mouse, Balb/c, male)	H22 hepatoma-mouse tumor-bearing model/HepG2 and HuH7	Oleuropein inhibits glycolysis by binding to GPI and may exhibit inhibitor-like effects on PYGM and PFKFB4. Consequently, it inhibits glycolytic metabolism, leading to an anti-tumor effect.	Disruption of redox homeostasis	Hong et al. [86]
**Oleuropein**	in vitro and in vivo (mouse, Balb/c nude, male)	22Rv1 xenograft tumor model/22Rv1	Surface-functionalized folate-targeted PEG liposomes loaded with oleuropein (OL-FML) exhibit anti-proliferative and apoptotic effects on prostate cancer cells. Additionally, enhanced tumor suppression and improved survival probability are observed in mice bearing 22Rv1-induced tumors when compared to the effects of oleuropein alone.	Inhibition of proliferation	Nassir et al. [87]
**Oleuropein**	in vitro	U251 and A172	Oleuropein reduces cell viability, triggers apoptosis, inhibits metastasis, and suppresses invasion in glioma cells by attenuating AKT signalling, concomitant with the elevation of Bax and the reduction in Bcl-2. Furthermore, it diminishes the expression of matrix metalloproteinase-9 (MMP-9) and MMP-2.	Inhibition of proliferation, metastasis, and modulation of cancer cell signalling pathways	Liu et al. [88]
**Oleuropein**	in vitro	A549, BEAS-2B	Oleuropein triggers apoptosis in lung cancer cells via the SOD2/O_2_⋅- /Akt/mGlo2 axis. This process involves the upregulation of mitochondrial Glo2 facilitated by the superoxide anion and the Akt signalling pathway.	Inducing cell death (apoptosis)	Antognelli et al. [89]
**Oleuropein**	in vitro	HEY and MCF-7	At a high dose, oleuropein inhibits the viability and growth of ovarian cancer cells by elevating reactive oxygen species (ROS) production and LIP levels, leading to the disruption of the cell cycle S-phase and the initiation of apoptosis. Conversely, at a low dose, oleuropein reduces ROS and LIP contents in ovarian cancer cells. Interestingly, this downregulation contradicts cell death induced by erastin-mediated oxidative stress.	Induction of cell death (apoptosis) and disruption of redox hemostasis	Scicchitano et al. [90]
**Oleuropein**	in vitro	Hela	Oleuropein reduces cell viability by inducing apoptosis, accomplished by suppressing anti-apoptotic genes such as Mcl1 and Bcl-2. Concurrently, it increases the expression of pro-apoptotic genes, such as Fas, Bid, TNFRSF10B, and the p53 tumor suppressor. Additionally, oleuropein increases the expression of miR-34a, miR-125b, and miR-29a while exhibiting decreased levels of miR-181b, miR-221, and miR-16.	Inducing cell death (apoptosis)	Amini-Farsani et al. [91]
**Oleuropein**	in vitro	MCF-7	Oleuropein reduces the migratory ability of breast cancer cells by inhibiting the epithelial-to-mesenchymal transition through the downregulation of sirtuin1 (SIRT1). Additionally, the simultaneous administration of oleuropein and doxorubicin amplifies their apoptotic and cytotoxic effects on these cell lines.	Inhibition of proliferation and migration	Choupani et al. [92]
**Oleuropein**	in vitro	AGS	Magnetic nano-oleuropein leads to the inhibition of gastric adenocarcinoma cell proliferation through the upregulation of KRAS and miR-200 gene expression.	Inhibition of proliferation	Barzegar et al. [93]
**Oleuropein**	in vitro	MCF-7, H1299, and HeLa	Oleuropein inhibits the growth of HeLa cells by inducing cell cycle arrest at the G2/M phase and promoting apoptosis. Its impact includes the upregulation of Bax and the concurrent downregulation of Bcl-2, potentially leading to the release of cytochrome c, which is associated with the mitochondrial apoptotic pathway. Additionally, oleuropein activates the JNK signalling pathway.	Inducing cell death (apoptosis)	Yao et al. [94]
**Oleuropein**	in vitro	MCF-7 and T47D	Oleuropein inhibits migration and invasion in ER-positive breast cancer by inducing autophagy through the upregulation of LC3II/LC3I and Beclin1 expression, along with a simultaneous downregulation of p62 expression.	Inhibition of migration and induction of autophagy	Lu et al. [95]
**Oleuropein**	in vitro and in vivo (mouse, BALB/c, male)	squamous cell carcinoma of the head and neck xenograft tumor mice/Tu686, 686LN-M2 and CAL-27	Oleuropein triggers apoptosis, reverses epithelial to mesenchymal transition (increase E-cadherin while reducing vimentin, snail, and MMP9), suppresses migration and invasion by suppressing the TGF-β1 signalling pathway. This inhibition affects the classical TGF-β1-Smad2 pathway and HIF-1α-related signalling in squamous cell carcinoma of the head and neck cells.	Inhibition of proliferation and inhibition of migration	Xu et al. [96]
**Oleuropein**	in vitro	SW480 and HEK293	Nanoparticles of iron oxide, coated with glucose and conjugated with oleuropein (Fe3O4@Glu-Oleuropein NPs), enhance the induction of necrotic and apoptotic cell death in colorectal cancer. Simultaneously, these nanoparticles lead to the down-regulation of GAS6-AS1, LINC00920, and FEZF1-AS1 long non-coding RNAs (lncRNAs). These genes are correlated with mutated KRAS and exhibit strong associations with hypoxia, KRAS signalling, DNA repair, and the IL-2/STAT5 signalling pathways.	Inhibition of proliferation	Niyaki et al. [97]
**Oleuropein**	in vitro	Tumor spheres/DLD-1 and 5-FU-resistant cells	Oleuropein inhibits the capacity for colorectal tumor sphere formation and triggers apoptosis. It also induces mitochondrial fragmentation and enhances mitochondrial superoxide production. Furthermore, the combination of oleuropein with 5-FU exhibits a synergistic effect in reducing colorectal cancer cells proliferation.	Inhibition of proliferation	Kim et al. [98]
**Oleuropein**	in vitro	U87	Oleuropein enhances cell viability, total oxidant capacity, and glutathione levels in glioblastoma cells following H_2_O_2_ administration. This suggests that oleuropein possesses strong antioxidative properties.	Antioxidative properties	Kucukgul et al. [99]
**Oleuropein**	in vivo (mouse. ICR female)	Mouse Skin Carcinogenesis	Oleuropein demonstrates a reduction in the thickness of epidermal hyperplasia when compared to the thick hyperplasia and epidermal disorganization observed in the DMBA/TPA control group. It also enhances apoptotic rates, as indicated by the activation of caspase-3, while concurrently decreasing the levels of MDA and GSH. Additionally, there is an increase in SOD levels. Oleuropein may function as a potential chemopreventive agent by exerting apoptotic and antioxidant defense activities during tumor development in skin carcinogenesis.	Inhibition of proliferation and antioxidative properties	Masre et al. [100]
**Oleuropein**	in vitro	MRC5, hMVECs and ECFCs	Oleuropein reduces migration of fibroblast SASP-dependent cells and tube formation of endothelial cells via the modulation of pro-angiogenic factor secretion surrounding of cell microenvironment.	Inhibition of migration and angiogenesis	Margheri et al. [101]
**Oleuropein**	in vitro	MCF-7	Nano-oleuropein enhances the Krebs cycle via upregulation of fumarylacetoacetase, succinate-CoA ligase, and isocitrate dehydrogenase1. Oleuropein functions by inhibiting glycolysis in cancer cells, prompting a shift towards the TCA cycle. This transformation may indicate inhibition of carcinogenesis in breast cancer cells.	Inhibition of proliferation	Kamrani et al. [102]
**Oleuropein**	in vivo (mouse, ICR mouse, female)	Mouse Skin Carcinogenesis	Oleuropein inhibits the progression of skin carcinogenesis through its antioxidant properties, reducing MDA levels while increasing GSH and SOD levels, and exerting an antiapoptotic effect on precancerous cells.	Inhibition of proliferation and antioxidative properties	John et al. [35]
**Oleuropein**	in vivo and in vitro (mouse, C57BL/6N, male)	Skin melanoma model/B16F10, Raw264.7, HUVECs and 3T3-L1	Oleuropein has inhibitory effects on the development of solid tumors and lymph node metastasis in C57BL/6 mice injected with B16F10 melanoma cells. Moreover, it directly impedes the differentiation of adipocytes. Additionally, dietary oleuropein restricts the accumulation of adipocytes and M2-MΦs induced by a high-fat diet (HFD). It also reduces the expression of VEGF-A, VEGF-D, and HIF-1α in tumor tissues. This suppression results in the inhibition of tumor angiogenesis and lymphangiogenesis in melanoma obese mice.	Inhibition of proliferation, inhibition of angiogenesis, and inhibition of metastasis	Song et al. [103]
**Oleuropein**	in vitro	HepG2	Oleuropein exhibits suppressive effects on paraquat (PQ)-induced oxidative stress in hepatocarcinoma cells by enhancing cell viability, normalizing β-tubulin expression levels, and reducing Casp-3. Furthermore, these protective effects mediated by oleuropein are linked to elevated malondialdehyde levels and increased superoxide generation.	Inhibition of oxidative stress	Katsoulieris et al. [104]
**Oleuropein**	in vivo (mouse, Balb/c male)	Balb/C	Oleuropein prevents tamoxifen from inducing oxidative DNA damage by decreasing the formation of 8-hydroxydeoxyguanosine.	Disruption of redox hemostasis	Jamshed et al. [105]
**Oleuropein**	in vitro and in vivo (mouse, Wistar, male)	Glioma cell implantation/C6	Oleuropein demonstrates the inhibition of glioma cell proliferation in vitro; however, it does not exhibit an anti-tumor effect on glioma tumors in vivo.	Inhibition of proliferation	Martínez-Martos et al. [106]
**Oleuropein**	in vitro	T-47D and MCF-7	Peracetylated derivatives of oleuropein demonstrate in vitro inhibitory effects on the proliferation of breast cancer cells and exhibit stronger antioxidant properties compared to free oleuropein.	Inhibition of proliferation	Bulotta et al. [107]
**Oleuropein**	in vitro	HepG2, HA22T, AML12 and HA59T	Oleuropein triggers cell cycle arrest, a process linked to the modulation of p53, p21, CDK1, and cyclin B1 levels. Additionally, oleuropein increases intracellular ROS levels while decreasing GSH levels. In HepG2 cells, oleuropein induces a rise of Ca^2+^ by releasing Ca^2+^ from the endoplasmic reticulum (ER) and facilitating Ca^2+^ influx through store-operated Ca^2+^ channels. Furthermore, oleuropein triggers Ca^2+^-associated cytotoxicity through ROS signalling and leads to cell cycle arrest.	Inhibition of proliferation	Cheng et al. [108]
**Oleacein and oleacanthal**	in vitro and in vivo (chicken chorioallantoic membrane (CAM))	Chicken chorioallantoic membrane and zebrafish caudal fin/BAEC, HeLa and HGF-1	Oleocanthal and oleacein inhibit the invasion, proliferation, and tube formation of endothelial cells, demonstrating an antiangiogenic effect. Furthermore, oleacein suppresses migration and induces apoptosis. Mechanistically, these compounds modulate signalling pathways associated with proliferation and survival, such as AKT and ERK1/2.	Induction of cell death (apoptosis), inhibition of angiogenesis, and modulation of cancer cell signalling pathways	Marrero et al. [109]
**Oleacein**	in vitro	501Mel	Oleacein induces cell growth inhibition in 501Mel melanoma cells through the induction of G1/S phase arrest, DNA fragmentation, and downregulation of antiapoptotic genes (BCL2 and MCL1) and proproliferative proteins (c-KIT, K-RAS, PIK3R3, mTOR). Moreover, oleacein increases the levels of miR-193a-5p (targeting PIK3R3 and mTOR), miR-193a-3p (targeting c-KIT, MCL1, and K-RAS), miR-34a-5p (targeting c-KIT and BCL2), and miR-16-5p (targeting K-RAS, BCL2, and mTOR) while decreasing miR-214-3p (targeting BAX).	Induction of cell death (apoptosis), inhibition of cell proliferation, and modulation of cancer cell signalling pathways	Carpi et al. [110]
**Oleacein**	in vitro	SH-SY5Y and WI-38	Oleacein inhibits the proliferation of neuroblastoma cells by inhibiting the cell cycle at the S phase and triggering apoptotic cell death. Apoptotic effect occurs markedly through upregulation of both p53 and Bax levels, coupled with a downregulation of Bcl-2 expression and STAT3 phosphorylation. Additionally, oleacein induces a decrease in migration and cell adhesion in these cells.	Induction of cell death (apoptosis), inhibition of migration, inhibition of cell proliferation, and modulation of cancer cell signalling pathways	Cirmi et al. [111]
**Oleacein**	in vitro	RPMI-8226, NCI-H929, MM1s, U266, and JJN3	Oleacein induces cell cycle arrest and apoptosis in multiple myeloma. It enhances the accumulation of α-tubulin and acetylated histones while concurrently reducing the expression of several class I/II histone deacetylases (HDACs) at both the protein and mRNA levels. The inhibition of HDACs by oleacein is linked to the down-regulation of Sp1, the primary transactivator of the HDACs promoter, through the activation of Caspase 8.	Inhibition of proliferation	Juli et al. [112]

## Data Availability

The raw data supporting the conclusions of this article will be made available by the authors on request.

## Supplementary Materials

The following supporting information can be downloaded at: https://www.mdpi.com/article/10.3390/nu16162755/s1, Supplementary Information S1: Description of search strategies applied for PubMed, Scopus and Web of Science databases.
