# Supplementary material for: Anticancer Effects of Secoiridoids—A Scoping Review of the Molecular Mechanisms behind the Chemopreventive Effects of the Olive Tree Components Oleocanthal, Oleacein, and Oleuropein
Source: Nutrients. 2024 Aug 18;16(16):2755. doi: 10.3390/nu16162755 (PMC11357637; doi:10.3390/nu16162755)
Supplement: Supplementary file 1 [file nutrients-16-02755-s001.zip › nutrients-3128841-supplementary.pdf]

Supplementary information S1.

Description of search strategies applied for PubMed, Scopus and Web of Science databases

PubMed 24/10/2023 → 1,432 articles

((((("oleocanthal" [Supplementary Concept]) OR ("OLE6 protein, Olea europaea" [Supplementary Concept] OR "OLE9 protein, Olea europaea" [Supplementary Concept] OR "OLE3 protein, Olea europaea" [Supplementary Concept] OR "olive leaf extract" [Supplementary Concept])) OR "Olive Oil"[Mesh])) OR (("Oleocanthal"[Title/Abstract] OR "Olea europaea"[Title/Abstract] OR "Olive leaf extract"[Title/Abstract] OR "Olive polyphenol"[Title/Abstract] OR "Olive"[Title/Abstract] OR "Olive oil"[Title/Abstract] OR "Extra virgin olive"[Title/Abstract] OR "Olive compound"[Title/Abstract] OR "Olive extract"[Title/Abstract] OR "Olive oil phenolic"[Title/Abstract]))) OR (("oleuropein" [Supplementary Concept] OR "10-hydroxyoleuropein" [Supplementary Concept] OR "Ligustrum"[Mesh]) OR (("Oleuropein"[Title/Abstract] OR "Olea europaea"[Title/Abstract] OR "Olive leaf extract"[Title/Abstract] OR "Olive polyphenol"[Title/Abstract] OR "Olive"[Title/Abstract] OR "Olive oil"[Title/Abstract] OR "Extra virgin olive"[Title/Abstract] OR "Olive compound"[Title/Abstract] OR "Olive extract"[Title/Abstract] OR "Olive oil phenolic"[Title/Abstract]) OR ("10-hydroxyoleuropein"[Title/Abstract] OR "Ligustrum"[Title/Abstract])))) AND (("Chemoprevention"[Mesh]) OR ("Chemoprevent*"[Title/Abstract] OR "Anticancer"[Title/Abstract] OR "Antitumor"[Title/Abstract] OR "Tumor prevent*"[Title/Abstract] OR "Cancer chemoprevent*"[Title/Abstract] OR "Cancer prevent*"[Title/Abstract] OR "Cancer"[Title/Abstract]))

Web of Science 24/10/2023 → 2,476 articles

((TS=(("oleocanthal" OR "OLE6 protein” OR “Olea europaea" OR "OLE9 protein” OR “Olea europaea" OR "OLE3 protein” OR “Olea europaea" OR "olive leaf extract" OR "Olive Oil" OR "Oleocanthal" OR "Olea europaea" OR "Olive leaf extract" OR "Olive polyphenol" OR "Olive" OR "Olive oil" OR "Extra virgin olive" OR "Olive compound" OR "Olive extract" OR "Olive oil phenolic"))) OR TS=(("oleuropein" OR "10-hydroxyoleuropein" OR "Ligustrum" OR "Oleuropein" OR "Olea europaea" OR "Olive leaf extract" OR "Olive polyphenol" OR "Olive" OR "Olive oil" OR "Extra virgin olive" OR "Olive compound" OR "Olive extract" OR "Olive oil phenolic" OR "10-hydroxyoleuropein" OR "Ligustrum"))) AND TS=(("Chemoprevention" OR "Chemoprevent*" OR "Anticancer" OR "Antitumor" OR "Tumor prevent*" OR "Cancer chemoprevent*" OR "Cancer prevent*" OR "Cancer"))

Scopus 24/10/2023 → 3,055 articles

TITLE-ABS-KEY ("oleocanthal" OR "OLE6 protein" OR "Olea europaea" OR "OLE9 protein" OR "Olea europaea" OR "OLE3 protein" OR "Olea europaea" OR "olive leaf extract" OR "Olive Oil" OR "Oleocanthal" OR "Olea europaea" OR "Olive leaf extract" OR "Olive polyphenol" OR "Olive" OR "Olive oil" OR "Extra virgin olive" OR "Olive compound" OR "Olive extract" OR "Olive oil phenolic") OR TITLE-ABS-KEY ("oleuropein" OR "10-hydroxyoleuropein" OR "Ligustrum" OR "Oleuropein" OR "Olea europaea" OR "Olive leaf extract" OR "Olive polyphenol" OR "Olive" OR "Olive oil" OR "Extra virgin olive" OR "Olive compound" OR "Olive extract" OR "Olive oil phenolic" OR "10-hydroxyoleuropein" OR "Ligustrum") AND TITLE-ABS-KEY ("Chemoprevention" OR "Chemoprevent*" OR "Anticancer" OR "Antitumor" OR "Tumor prevent*" OR "Cancer chemoprevent*" OR "Cancer prevent*" OR "Cancer")
